# Adaptation of an Adult Web Application for Type 1 Diabetes Self-management to Youth Using the Behavior Change Wheel to Tailor the Needs of Health Care Transition: Qualitative Interview Study

**DOI:** 10.2196/42564

**Published:** 2023-04-26

**Authors:** Li Feng Xie, Asmaa Housni, Meranda Nakhla, Rosemarie Cianci, Catherine Leroux, Deborah Da Costa, Anne-Sophie Brazeau

**Affiliations:** 1 School of Human Nutrition McGill University Sainte-Anne-de-Bellevue, QC Canada; 2 The Research Institute of the McGill University Health Centre Montreal, QC Canada; 3 Division of Endocrinology, Montreal Children's Hospital Montreal, QC Canada; 4 Department of Medicine McGill University Montreal, QC Canada; 5 Montreal Diabetes Research Center Montreal, QC Canada

**Keywords:** type 1 diabetes, youth, eHealth, self-management, mobile phone, peer support

## Abstract

**Background:**

Youth (aged 14-24 years) living with type 1 diabetes (T1D) encounter increased challenges in their diabetes self-management (DSM), especially during the transition to adult care. Although DSM education and support are imperative, there is insufficient information on how web-based digital tools tailored to their demands can be developed.

**Objective:**

On the basis of the Behavior Change Wheel, this study aims to identify, among youth living with T1D, the needs and factors influencing their DSM in the context of health care transition and to inform the adaptation (content and features) of an adult self-guided web application (*Support*).

**Methods:**

Internet-based semistructured individual interviews based on a phenomenological study design were conducted with 21 youths, and transcripts were analyzed using an inductive approach with concept mapping.

**Results:**

Factors influencing T1D self-management were categorized into barriers and facilitators and then as external or internal. Features influencing the accessibility to information, increasing the sense of support, and use of the tool were positively accepted. Features unrelated to their expectations of digital tool use or difficulty navigating were viewed negatively. Participants expressed an interest in reliable, practical, and novel educational content. Although youth considered the information provided by medical professionals to be important, peer exchange was deemed necessary to obtain a practical perspective and real-life examples.

**Conclusions:**

Compared with the adult population, in addition to tailored content and a simplified information search process, when building a DSM education and support digital tool for youth, features should be selected to encourage supervised peer exchange.

## Introduction

### Background

In Canada, the estimated prevalence of people living with type 1 diabetes (T1D) was 276,284 in 2021, including 31,601 people aged <19 years [1]. T1D is an autoimmune disease that requires external insulin injection and sustainable diabetes self-management (DSM) behaviors to maintain optimal glucose levels [2]. On the basis of an American survey of certified diabetes educators, a child diagnosed with T1D needs 78-305 minutes daily to complete all the recommended components of DSM [3]. Given this intensity and lifelong efforts, adherence to medical treatment is difficult to achieve, especially for adolescents and young adults with T1D (aged 14-24 years) [4]. The adolescent period is characterized by physiological changes (eg, an increase in insulin resistance), navigating social constructs, peer influence, a shift in family dynamics, and the transfer of responsibilities from parent to child [5]. For instance, in the province of Quebec, Canada, adolescents aged ≥14 years can consent to care alone if there is no serious risk to health [6] and make their own decisions in their diabetes management. Despite the varied T1D management priorities across early adolescence (eg, aged 14 years) and young adulthood (eg, aged 24 years) [7], both groups are challenged with the transition in care. Although parents might still play a central role and participate in the transition process [8], youth, as they age, are searching for more diabetes autonomy and emancipation from their family [9,10]. This health care transition period can thus be viewed as an opportunity to equip youth with the necessary education. Furthermore, pairing them with peers who recently experienced the transition (eg, those aged ≥18 years) can support them in acquiring DSM behaviors and addressing these additional responsibilities [11].

One approach to increasing the acquisition of DSM behaviors among youth living with T1D is the use of self-guided (ie, absence of individual contact with health care professionals [HCPs]) digital tools. Web-based approaches can be interesting given the lower development cost compared with a mobile-based tool [12] and the possibility of designing web apps to be mobile responsive. Studies have shown that youth are active users of digital health technologies, and they appreciate using web-based information as an opportunity to improve their health and use social media as an emotional support [13]. However, there is a gap in resources specially developed for this population [13] and limited information on how their needs can be linked to behavior change theories and translated into DSM education and support (DSME/S) interventions. For instance, Diabetes Youth Empowerment and Support is a 12-week self-guided web-based program developed for young adults (aged 18-35 years) in Australia [14]. Regardless of its acceptability among the target population and inclusion of topics related to transition, the intervention only addressed people who were at the end of the health care transition (eg, those aged ≥18 years) and did not prepare adolescents for the transition. In addition, the development of this intervention was not guided by behavior change theories or frameworks. Another study focused on a younger population aged 12-16 years living with T1D, but the designed mobile app was related mainly to the tracking of blood glucose values, and the challenges of health care transition were not addressed [15].

The integration of behavior change theories or frameworks such as the Behavior Change Wheel (BCW) guides the understanding of behavior mechanisms and can increase the applicability of an intervention in the real-world setting [16,17]. The BCW starts with the Capability-Opportunity-Motivation-Behavior model at its core to understand a behavior and is then encircled by 9 intervention functions needed to change behaviors; such interventions are supported by 7 policy categories [18]. The BCW also links these interventions to behavior change techniques (BCTs), which are the backbone of each behavior change intervention [19]. The integration of BCTs within interventions can potentially increase their replicability and the positive outcomes of behavioral changes. To convey these behavioral changes from a technological perspective, BCTs can guide the choice of characteristics and features [19,20]. An example of the application of BCTs for T1D is *Support* [21], a self-guided web app for people living with T1D that has an evidence-based design [22]. It offers multidimensional education and support to adults living with T1D to improve their DSM. Developed by HCPs in the field, in collaboration with patient partners, it is personalized based on the user’s treatment regimen to offer tailored content. This web app also includes a discussion forum mediated by HCPs and features requiring active participation (eg, quizzes and calculators) [21]. However, as youth living with T1D encounter specific challenges of health care transition, DSME/S for this population should address this specific issue.

### Objectives

Considering the lack of accessible self-guided web apps based on youths’ interests and needs [23,24], as an early exploratory developmental study based on the BCW, this study aimed to identify, among youth living with T1D, the needs and factors influencing their DSM in the context of health care transition and to inform the adaptation (content and features) of an adult self-guided web app (*Support)* to those needs.

## Methods

### Study Design and Recruitment

We conducted a phenomenological qualitative study (ie, a study focusing on the experience of the participants related to their DSM in the context of health care transition and their interest in using digital health tools for DSM) with semistructured individual interviews. The method section follows the Consolidated Criteria for Reporting Qualitative Research checklist [25].

We recruited participants from 2 age categories (14-18 years to understand the needs of people who are preparing for health care transition and 19-24 years to understand the experience of people who have recently transitioned to adult care from pediatric care). The inclusion criteria were being aged between 14 and 24 years; living in the province of Quebec, Canada; diagnosed with T1D; and being able to communicate in English or French. The recruitment followed three main convenience and purposive sampling methods: (1) word of mouth; (2) email invitation via the BETTER (Behaviors, Therapies, Technologies and Hypoglycemic Risk in Type 1 Diabetes) registry (a registry of people living with T1D in Quebec; ClinicalTrials.gov identifier: NCT03720197 [26]); and (3) advertisement on social media (eg, private T1D Facebook groups) and on the study website. Potential participants were screened for eligibility via phone by a research assistant. Written informed consent was obtained by email before the interview, which was conducted using Microsoft Teams.

### Ethics Approval

The study was approved by the McGill University Research Ethics Board (20-08-036).

### Semistructured Interview

The interviews were planned to last 60 minutes and were conducted in French or English. The participants had the option to turn on their cameras or proceed with voice only. All interviews were recorded and then transcribed. The interviews followed a guide developed by a multidisciplinary team (dietitians, nurses, endocrinologists, and pediatricians) and were reviewed and tested by patient partners (Multimedia Appendix 1). The interview guide was adapted (eg, modifying the wording and converting a few closed-ended questions to open-ended questions) after 4 interviews. All interviews were led by a female research assistant (CL, a registered dietitian with experience in clinical diabetes care). The participants did not know their interviewer before the study.

The interviews were based on an existing self-guided web app designed for adults with T1D (ie, *Support* [21]) as an example of a web-based DSME/S resource. The information on *Support* is divided into 6 learning categories with 3 levels of complexity. The web app integrates various features to facilitate the learning and navigation experience (eg, a discussion forum facilitated by an HCP, quizzes, and videos). This web app is completely self-guided, and users can learn at their own pace. Before each interview, participants received a PowerPoint presentation of the adult *Support* and a 3-minute explanatory video of the web app. At the beginning of each interview, the research assistant confirmed that the participants had the opportunity to review the material and asked if they had any related questions. If the participant did not review the material, the interviewer presented it at the beginning of the interview.

The interview consisted of 4 sections with a total of 20 questions (including open- and closed-ended questions). Interviews started with a self-introduction of the participants, followed by their current diabetes management practices (eg, their treatment plan [eg, type of insulin use and method of blood glucose monitoring], where they find information related to health and diabetes, and their confidence in managing diabetes) and their general use of web-based education tools. The interviewer then probed for feedback regarding *Support* (eg, most preferred features and adaptations that should be made for youth) and the potential for creating new content (eg, by providing examples of barriers in daily life and other topics that they would like to discuss), and the interviewer then concluded the interview. Some of these questions included probes that facilitated the discussion if participants initially had no answers.

Upon completion of the interview, each participant received a CAD $40 electronic gift card (US $31) to compensate for their time. Each participant was interviewed once and was also invited to send their comments or suggestions by email following their interview, if applicable. However, no comments were received after the interviews.

### Transcript and Analysis

The interviews were first transcribed by 1 of the researchers (LFX or AH) and then reviewed by the other researcher to confirm the accuracy of the information. Two researchers (LFX and AH) performed the coding independently using NVivo software (QSR International) and discussed the agreement on the coding attributed to each transcript section. The researchers reached mutual agreement for all codes included. The percentage of agreement was calculated based on the total number of included codes divided by the largest number of codes independently found by the 2 researchers. The interviews were analyzed using an iterative inductive approach. The initial codes were determined based on 2 randomly selected interviews and then adapted throughout the analyses. Inductively, codes with similar topics related to their DSM or feedback regarding *Support* were then merged into categories and further into themes using concept mapping. All analyses were performed in French. Codes and quotes were translated into English using a forward-backward translation process by 3 bilingual researchers (LFX, AH, and RC) for the purpose of publication. Results were discussed with research patient partners but were not returned to the participants.

## Results

### Overview

Among the 32 invitation emails sent, 27 potential participants contacted the research assistant and were screened for eligibility. Among the eligible participants, 22 agreed to participate, but 1 participant was excluded during the interview because of difficulty in understanding the questions and responding to them. The final sample size included 21 participants: 8 participants in the 14-18 years category (5 women and 3 men) and 13 participants in the 19-24 years category (7 women and 6 men). The mean diabetes duration was 9.4 (SD 4.5) years, ranging from 1 to 16 (median 10, IQR 5.1) years; 86% (18/21) of the participants were White. Most participants were using an insulin pump (16/21, 76%) and continuous or flash glucose monitoring systems (15/21, 71%). One participant (Participant 5, man, aged 23 years, 17 years since diagnosis) used *Support* for 6 months before the interview. The interviews were conducted between October 2020 and January 2021. The mean length of the interviews was 44 (range 27-62) minutes.

**Figure 1:**
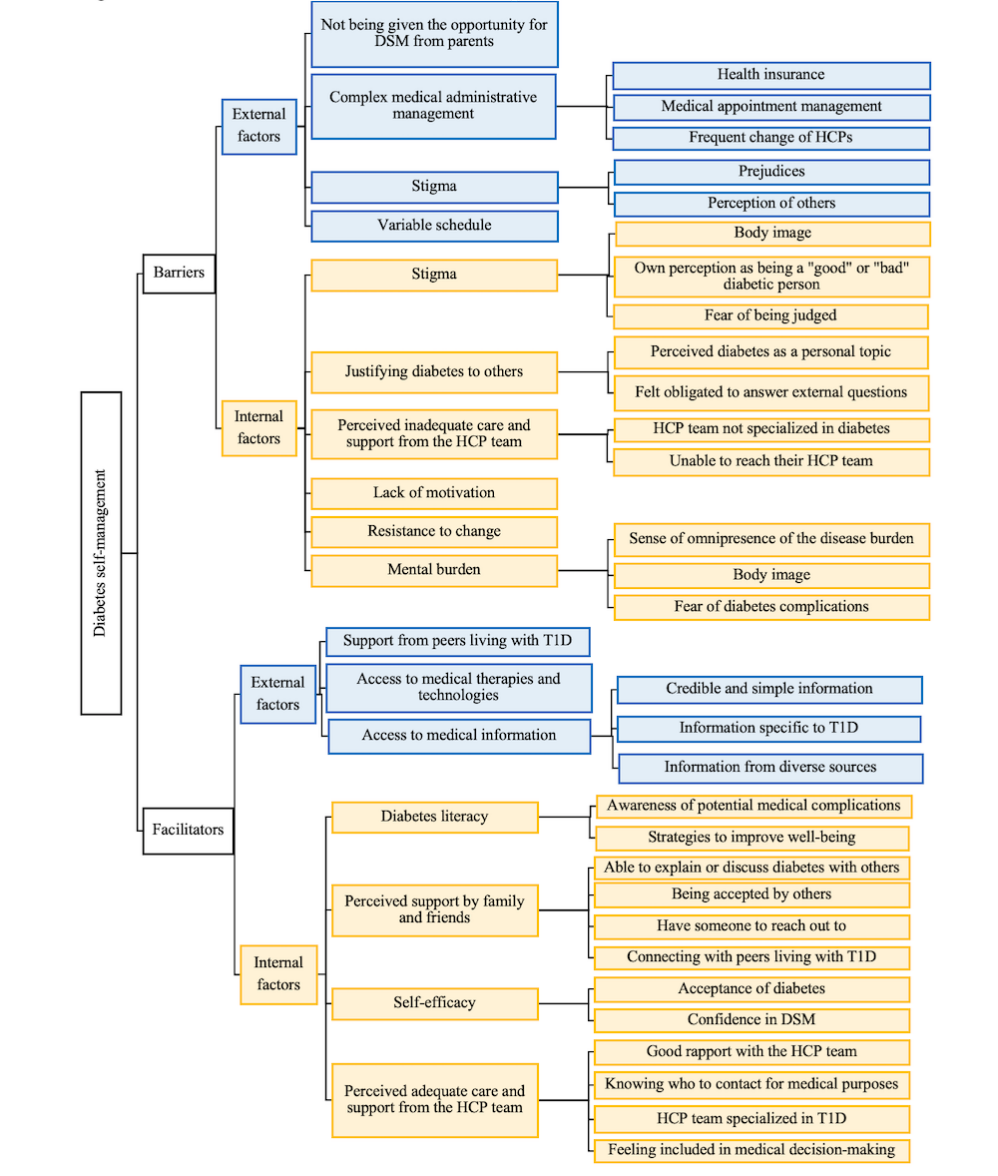
Barriers and facilitators of diabetes self-management (DSM). HCP: Healthcare professional; T1D: Type 1 diabetes.

Data saturation (ie, no new codes were determined during the analysis) was reached after analyzing 17 transcripts, and the average agreement score was 72%. Codes related to DSM were categorized into themes using a concept mapping approach (Figure 1), and feedback regarding the features was analyzed (Tables 1and 2).

**Table 1:** Examples of quotes and discussed features starting with A-P.

Characteristics or features	Related behavior change techniques	Description	Feedback^a^	Examples of quotes
Anonymity	Avoid aversive stimulus^b^	Ability to use the tool without being known by others	(+) Access to information Sense of support	“They [youth with T1D] are afraid to confess to others [peers or health care professionals]. It might be important for them to use these platforms. Like knowing the information without others knowing about it” (Participant 12, male, aged 14 years, 6 years since diagnosis).
Carbohydrate calculator	Avoid aversive stimulus Problem-solving	Calculator to estimate the food’s carbohydrate content	(+) Access to information	“I was wondering if it would be possible if you have an app that when taking a photo, it would be able to estimate your carbs” (Participant 15, male, aged 21 years, 6 years since diagnosis).
Cartoon illustrations	Avoid aversive stimulus	General visual design of *Support* (eg, pictures, colors, and word font)	(+) Access to information Sense of support (−) Dislike owing to personal preference Not perceived relevant for DSM^c^	“Then the site is also visually beautiful, I have the impression that since I find that it is interesting, it can motivate me to go on it” (Participant 10, female, aged 19 years, 3 years since diagnosis). “[...] I found that it [the profile avatars] was a little bit childish. Maybe I would have put it a little more suited because it is a clientele over 18 years old” (Participant 15, male, aged 21 years, 6 years since diagnosis).
Categories	Avoid aversive stimulus	Learning modules classified in categories	(+) Access to information	“The ease of finding information, with it clearly divided into sections. If the first time I had difficulty finding the answer to my question, for example, in relation to food, I would be less inclined to go to this site” (Participant 16, female, aged 19 years, 9 years since diagnosis).
Chat room	Social support	One-on-one message with a health care professional or another participant	(+) Access to information Sense of support	“I would say drugs and alcohol are not the most comfortable topic. [...] I think it’s more comfortable to talk about it anonymously, or in a chatroom, than it is face-to-face with your doctor, especially as a 15, 16, 17-year-old. [...]” (Participant 19, male, aged 16 years, 9 years since diagnosis). “I don’t know if that would be possible, but sometimes, on sites, there are little chats, something where if you have a question [...] you sometimes contact someone who is there directly” (Participant 5, male, aged 23 years, 17 years since diagnosis)
Discussion forum	Social support	A health care professional–moderated forum where users can ask questions or post comments	(+) Access to information Engagement with the tool Sense of support (−) Difficulty using the feature Dislike owing to personal preference Not perceived relevant for DSM	“Also, the exchange blog, because sometimes yes there are professionals, but they can’t really feel 100% what it is like as a diabetic. So sometimes having support from other people who aim for the same things as you, [...] it’s more reassuring I would say” (Participant 18, female, aged 16 years, 15 years since diagnosis). “[I liked] the discussion forums, […] I could find them everywhere else; the only difference is that they are not like moderated by a health professional, but you know a lot of social networks have people sharing their experience, there is even a social network that was created just for diabetics” (Participant 13, female, aged 16 years, 5 years since diagnosis).
Downloadable summary documents	Credible source Avoid aversive stimulus	Summary documents that are ready to be downloaded and printed	(+) Access to information	“I also like when there are, let’s say, cheat-sheets, or whatever, that you can print out and keep with you, not necessarily to have to look for them on the site, that also helps a lot” (Participant 18, female, aged 16 years, 15 years since diagnosis).
Gamification	Nonspecific reward	Accumulation of points, trophies, and certificates; provide possibilities of having competitions among participants	(+) Access to information Engagement with the tool Sense of support (−) Dislike owing to personal preference Not perceived relevant for DSM	“We accumulate them [points] on the different categories. It’s fun. [...] I find it must be like a kind of self-fulfillment feeling, you say to yourself, I’m a good person” (Participant 14, female, aged 16 years, 5 years since diagnosis). “Maybe if I had been younger, that I just got diagnosed with diabetes, that would motivate me more, but now it’s been 10 years that I have it, so I learned to manage well. So, whether I have a trophy or not, it won’t influence me to change my control” (Participant 17, female, aged 23 years, 10 years since diagnosis).
Internal search engine	Avoid aversive stimulus	Search engine by keywords	(+) Access to information	“You forget something, you want to remember, you are going to look for the specific information, but this is found in lesson 3, you have to do all the lessons before” (Participant 3, male, aged 22 years, 6 years since diagnosis).
Links to external resources	Social support	External resources provided (eg, from governmental and health institutions) within the digital tool	(+) Access to information	“[It would be useful to have information] which is not necessarily on the platform, but links to other external links or phone numbers” (Participant 18, female, aged 16 years, 15 years since diagnosis).
Module duration display	Avoid aversive stimulus	Estimated time needed for completion	(+) Access to information Engagement with the tool	“When you see that it's 10 minutes, it motivates you a little to finish the class” (Participant 21, male, aged 24 years, 10 years since diagnosis).
Notifications	Avoid aversive stimulus	Update notifications of the app, news, or DSM	(+) Access to information Engagement with the tool	“Maybe there should be a notification to say that there is some news that came out. But I’m not sure people have, let’s say, the habit to log in once a week just to go and see if there is any news” (Participant 15, male, aged 21 years, 6 years since diagnosis).
Personal objectives	Commitment Goal setting Review behavior goal	Self-given or provided personal objectives for DSM	(+) Engagement with the tool	“Maybe weekly goals to achieve. Like maybe ‘Have you managed to measure your blood sugar a certain number of times?’, goals to achieve which really makes you want to do it” (Participant 21, male, aged 24 years, 10 years since diagnosis).
Placement quiz	Graded tasks	Diabetes-related questions to adjust the learning at beginning of the program	(+) Access to information	“Maybe it could be [helpful] to take a little test to establish the level and know […] at what course we should be placed at” (Participant 13, female, aged 16 years, 5 years since diagnosis).
Progress visualization	Self-monitoring of behavior	Timeline within the platform to see the learning progress and option to exit the learning modules at any time with the progress saved	(+) Access to information Engagement with the tool	“There are little points that show our progress in the program, I find that relevant because […] it’s a visual cue. It tells us we’re about halfway” (Participant 16, female, aged 19 years, 9 years since diagnosis). “I also found it fun that you could see your progress so that you don’t have to do [the course] all at once. You can do part of it, and come back, you know where you’ve been, […] it’s visual” (Participant 20, male, aged 19 years, 11 years since diagnosis).

^a^The (+) sign refers to positive feedback regarding the presence of features or characteristics on the platform for the diabetes self-management of the participants, and the (−) sign refers to negative feedback. A feature or characteristic can have a mix of positive and negative feedback.

^b^“Avoid aversive stimulus” is adapted from the “remove aversive stimulus” of the behavior change techniques taxonomy.

^c^DSM: diabetes self-management.

**Table 2:** Examples of quotes and discussed features starting with Q-Z.

Characteristics or features	Related behavior change techniques	Description	Feedback^a^	Examples of quotes
Quiz	Feedback on outcomes of behavior	Questions for participants throughout the learning modules; correct answers are provided right after their answer	(+) Access to information Engagement with the tool (−) Dislike owing to personal preference	“I think the quizzes are good, I think they’re important just to make sure you know what you’re doing so that you don’t get in trouble when you’re actually dealing with stuff. Like, just basic stuff in the quizzes” (Participant 19, male, aged 16 years, 9 years since diagnosis). “Personally, the quizzes appeal to me a little less, but at the same time, I tell myself that there may be people for whom it is easier to know that they have understood the material” (Participant 18, female, aged 16 years, 15 years since diagnosis).
Sharable link	Credible source Social support	Automatically generated sharable link	(+) Sense of support	“Maybe articles they can be shared on social media [...]., Like if there is a way to share an article that discusses a particular topic on Facebook [...] with everyone and say: Take 2 seconds of your day to read this” (Participant 5, male, aged 23 years, 17 years since diagnosis).
Smartphone compatibility	Avoid aversive stimulus	Ability to navigate using a smartphone	(+) Access to information	“In the format of an application, [...] it appeals to me a lot more in my cellphone, since I already have my sensor, everything is in there” (Participant 17, female, aged 23 years, 10 years since diagnosis).
Tangible rewards	Material incentive	Rewards (eg, pen and booklets) given based on the points	(+) Engagement with the tool	“Maybe a way to attract teenagers more, [...], but also like having small prizes, but physical ones, then it could be a Dex4 package [...] or pencils. [...] I think that it might motivate” (Participant 13, female, aged 16 years, 5 years since diagnosis).
Testimonials	Credible source Identification of self as a role model Social support	Stories from people living with type 1 diabetes	(+) Engagement with the tool Sense of support	“People giving their experiences, and showing, demonstrating to people that we are all different, and that each body reacts differently, [...] it’s really just learning to know how the body reacts in relation to it all” (Participant 14, female, aged 16 years, 5 years since diagnosis).
Unrestricted access to all the modules	Avoid aversive stimulus	All the learning modules are unblocked initially and free of access	(+) Access to information	“Another suggestion would be keeping the order of the modules but leave them unblocked, [...] if you don’t have a lot of knowledge in diabetes, it can be useful to start with the basics” (Participant 3, male, aged 22 years, 6 years since diagnosis).
Videos	Credible source Avoid aversive stimulus	Information given in a video format	(+) Access to information	“I think videos are more relevant for learning purposes away from an academic context. I think it’s easier for people to learn by video than by written stuff, the concentration is different I find” (Participant 1, female, aged 22 years, 19 years since diagnosis).

^a^The (+) sign refers to positive feedback regarding the presence of features or characteristics on the platform for the diabetes self-management of the participants, and the (−) sign refers to negative feedback. A feature or characteristic can have a mix of positive and negative feedback.

### Understanding Needs for DSM

#### Overview

Participants expressed their experiences and needs with DSM in the context of health care transition. The information was grouped into barriers and facilitators to DSM and further categorized into external (ie, factors that participants could not alter on their own) and internal factors (ie, factors that participants could alter on their own; Figure 1). Except for the factor “not being given the opportunity for DSM from parents,” which was only mentioned in the 14- to 18-years groups, all the other factors were covered in both age groups. Examples of the quotes considered for each code are provided in Multimedia Appendix 2.

#### External Barriers

Four external barriers were identified (Figure 1). With adolescents gradually acquiring more autonomy, 1 external barrier they faced was not being given the opportunity from their parents to self-manage their diabetes. One youth described it as feeling “handicapped” (Participant 10, female, aged 19 years, 3 years since diagnosis). This prevented them from managing their diabetes autonomously, given the involvement of their parents in their diabetes care. Autonomy also brings more responsibility, such that youth slowly take over the administrative aspects of their diabetes, such as making medical appointments and dealing with health insurance. Participants also cited the transition process as a barrier, including transfer to a new health care team and setting. Administrative factors were described as complex and as barriers to adequately managing T1D. A variable schedule was also stated as adding difficulty to DSM at this age. This affected their sleep, exercise, and meal patterns, which had a magnified impact on diabetes management. One participant said the following:

Basically everything can affect your blood sugar is how I see it. Like stress, eating, sleep [...] anything can, which is really hard.

Participant 19, male, aged 16 years, 9 years since diagnosis

Stigma was also a barrier and was expressed to be both external and internal. The prejudices and perceptions of others were external stigmas experienced by participants. It ranged from the misconception that sugar intake was alone responsible for diabetes development, wrongful associations between insulin injection and drug use, and discrimination in their capability to perform actions to stereotyping the body weight of “a diabetic [person].” One participant shared the following:

I have often been told: “I do not understand why you are diabetic, you seem to eat well and you don’t seem to be very overweight.” No, but it’s not that.

Participant 18, female, aged 16 years, 15 years since diagnosis

#### Internal Barriers

Stigma can also be an internal barrier. In fact, participants expressed concerns about their body image and internalized behavior labeling such as considering themselves as a “good” or “bad diabetic [person].” (Participant 21, male, aged 24 years, 10 years since diagnosis). In addition, youth’s fear of being judged by others prevents them from properly managing their diabetes if they perceive it to be a “burden” for others. For example, one of the participants said the following*:*

I will wait until the end of the class to eat something. Otherwise, people will look at me as if I were sick [...]. So, it happened to me to wait for class to end to treat hypos.

Participant 3, male, aged 22 years, 6 years since diagnosis

In addition, the difficulty of navigating the high expectations from their health care team was expressed by a participant and can prevent suitable health care support:

To understand that […] we are not perfect patients, who take their blood sugar on time, and then they eat a certain number of grams of carbohydrates.

Participant 21, male, aged 24 years, 10 years since diagnosis

Participants also expressed their observations related to the lack of specialization in T1D care transition from pediatric to adulthood and their apprehensions of being left alone. One youth shared the following:

It was like a shock to me, because [my new health care team] was supposed to be medical specialists; but they had no expertise in diabetes technology at all. Then he didn’t even look at my blood sugar.

Participant 3, male, aged 22 years, 6 years since diagnosis

Another internal barrier was feeling the need to justify diabetes to others and to answer continuous inquiries from others. In fact, some participants perceived diabetes as a personal topic and preferred not to spend more time discussing it with others. In contrast, it was important for them to feel heard of and supported by the people around them.

The lack of motivation was expressed by some participants as a lack of interest and a lower priority placed on their DSM. This was mainly related to the difficulty of dealing with and accepting their diabetes. One participant said the following:

I wanted to hear […] the stuff that might help people with diabetes acceptance and take responsibility [...]. Talk about it a little more when I was young, to have cues to deal with diabetes […] to be able to explain it without living in too much discomfort.

Participant 17, female, aged 23 years, 10 years since diagnosis

This becomes an even greater challenge when coupled with the mental burden. Concerns were expressed regarding body image and difficulty with weight, a sense of omnipresence of the diabetes burden, and the fear of consequences related to T1D that adds to the burden of this condition.

The final mentioned internal barrier was the resistance to change. The difficulty of breaking behavior and acquiring a new way of managing their diabetes encompassed the struggles of maintaining a habit. In fact, in addition to understanding and knowing how to deal with certain aspects of diabetes, consistency in performing these actions is an issue. One participant said the following:

I know how to calculate my carbohydrates, I know everything to do, but sometimes it’s to take the initiative, calculate […] it’s more doing it than knowing it.

Participant 18, female, aged 16 years, 15 years since diagnosis

#### External Facilitators

Several facilitators were voiced by the participants as opportunities to strengthen their DSM, such as connecting with peers living with T1D to share their daily lives and routine. Their peers are also a source of practical information, a participant shared the following:

[The doctor] doesn’t live the same reality as me, [and I would be more interested] to see how people can apply it, sometimes it helps me when I meet a diabetic person.

Participant 17, female, aged 23 years, 10 years since diagnosis

Other external facilitators included the use of medical technologies and therapies and access to medical information. One participant shared that consulting a resource such as *Support* was interesting because it was “a way to acquire information more easily, more quickly, because an appointment with an endocrinologist is long” (Participant 12, male, aged 14 years, 6 years since diagnosis).

#### Internal Facilitators

Participants’ knowledge of technology use contributed to their diabetes literacy and facilitated DSM. One participant expressed her enthusiasm saying the following:

I am very excited! I can know how long it has been since I injected my last dose [using an insulin pump therapy].

Participant 4, female, aged 24 years, 10 years since diagnosis

Although acquiring strategies to improve well-being was deemed important for youth’s DSM, being aware and understanding complications and their breadth of impact on their health and lives were central to facilitating DSM, as a youth inquired:

If I didn’t inject, what would it do? At 10 months of diabetes, I still don’t even know what it [the consequences of not injecting diabetes] does [...].

Participant 11, female, aged 16 years, 1 year since diagnosis

According to the participants, adequate DSM is closely linked to perceived support (from their families, friends, and HCPs), as this can facilitate their communication with others about diabetes, being accepted by others beyond their health condition, and having someone to reach out to if they ever feel the need to.

### Factors Impacting the Acceptability of a Self-management App’s Features

#### Overview

Participants individually proposed a list of potential features or characteristics included in the self-guided DSM digital tools based on preexisting features in *Support* (Tables 1 and 2). According to the participants, a feature or a characteristic tends to be positively accepted if it (1) provides access to the information, (2) increases a sense of support, and (3) increases engagement with the web app. They were viewed negatively mainly because of (1) personal preferences, (2) difficulty using the feature, and (3) perceived irrelevance to DSM. A feature may be associated with one or more of these factors.

#### Accessibility to the Information

A feature increases the accessibility to the information when it facilitates autonomous web app navigation by the participants, organizes the content in a logical and simple way, or adds flexibility to their learning process. Features such as an internal search engine, downloadable PDF documents, and specific module categories were all considered as methods to organize information in a simplistic manner and facilitate navigation on the web app. The participant who used *Support* for 6 months found it difficult to navigate without an internal search engine. He stated the following:

there was one [module] I wanted to go see and then I was like oh my God where is it. I had to scroll, look a bit through the pages [...] I think [a search engine] can be handy.

Participant 5, male, aged 23 years, 17 years since diagnosis

Therefore, implementing features to help users save time should be considered as one of the main priorities during the design of the platform.

To add flexibility to the learning experience, participants suggested that features such as smartphone compatibility should be considered, as viewing the platform on a phone (web page or app-based) is more convenient than opening a browser on a computer.

#### Sense of Support

Sense of support refers to the need for youth to not feel alone in their diabetes management. This idea includes being able to communicate diabetes-related information with people who do and who do not have this condition. This can be realized through a discussion forum, chat rooms, or the incorporation of testimonials. One participant mentioned the following:

For young adults, we are more and more focused on the connection with others, the discussion, the socializing on networks.

Participant 2, female, aged 23 years, 14 years since diagnosis

Communication with others helps them understand that others are in the same situation and that there is not only one solution to issues:

[Having] people giving their experiences, then showing or demonstrating to people that we are all different, that each body reacts differently […], it’s really just learning to know how the body reacts with regards to it.

Participant 14, female, aged 16 years, 4 years since diagnosis

#### Use of the Web App

Participants discussed how the choice of features could impact their use of the web app. For instance, a visually appealing platform can increase their motivation and curiosity to learn and encourage them to return. Displaying the progress of the module completion was also seen to make users feel accomplished, and setting personal objectives may increase their desire for knowledge application, further reinforcing their learnings. Notifications can help increase the use of the digital tools by reminding people of their existence and informing users about new content.

Other features that have a potential impact on engagement include rewards, but the opinions were divided. The integrated gamification (eg, trophies, certificates, and quizzes) may benefit some participants by keeping them using the tool:

I think that can be a motivator and make me feel proud. Like I got my new trophy […], basically, it can be a personal pride.

Participant 12, male, aged 14 years, 6 years since diagnosis

However, for others, it would have no impact on their use:

I think that [trophies] don’t matter to me, [...] I would like it for the younger people.

Participant 5, male, aged 23 years, 17 years since diagnosis

#### Characteristics Leading to Negative Acceptability

In addition to not perceiving a feature as being useful for its intended goal, the difficulty of using a feature can also be a barrier. For instance, the discussion forum received mixed feedback owing to its current format (ie, under a specific tab and participants needed to click on each topic to investigate the posts):

Well maybe a different format, [...] because I have the impression that a forum is good for asking your own questions, but you lose some of the information because you don’t tend to look at [the answer of other posts].

Participant 10, female, aged 19 years, 3 years since diagnosis

Therefore, it was suggested to display all the posts in a chronological order and have them automatically shown on their dashboard. The discussion forum could also be directly integrated into a social media platform (eg, Facebook), as many youths are already using it.

Personal preferences were the third explanation given by the participants regarding the negative acceptability of the features. This is reflected in comments on the design of the web app (eg, considering cartoon illustrations as childish) or related to their experience (eg, associating quizzes with academic performance). Participants specifically highlighted that they do not feel “like reading huge paragraphs and then answering quizzes again [...] after a day of school” (Participant 16, female, aged 19 years, 9 years since diagnosis). One of the proposed solutions is to increase the use of videos as they “are lighter, as it is more like listening to a show” (Participant 22, male, aged 22 years, 13 years since diagnosis).

### Suggestions for Diabetes Education Content

On the basis of the existing educational content provided to participants, a list of additional topics was discussed and is provided in Multimedia Appendix 3. The results highlighted the characteristics of the learning content that will be the most appreciated by the participants: (1) reliable, (2) practical, and (3) novel.

#### Reliability of the Educational Information Provided

The source of reliability of information differs for medical (eg, understanding the impact of alcohol on glycemic control) and experience-related topics (eg, how to limit alcohol consumption at a party). For medical information, a high level of reliability would be the ones sourced from governmental or organizational websites, magazines, or journals:

I’m really looking for [...] something reliable. Either by the government or anything, such as a project or a foundation that is relatively reliable.

Participant 7, female, aged 17 years, 14 years since diagnosis

Participants questioned the reliability of the information from discussion forums and social media group pages. Their reliability, or potential lack thereof, is a barrier for participants seeking information using these tools. However, this issue of credibility could be resolved with the supervision of an HCP who would address invalid recommendations:

It’s true that having a forum with specialized [health care professionals] would be a real bonus because on the Internet we really get advice that we think we can follow [but they are] not given by professionals.

Participant 21, male, aged 24 years, 10 years since diagnosis

Although the role of HCPs and information coming from credited references were essential for medical advice, this appeared to be lessened when referring to personal experience–related information. One participant mentioned the following:

Testimonials [...] [are] still pleasant, [...] we see that we are not all alone. In the same boat, there are also others who have the same problem.

Participant 4, female, aged 24 years, 10 years since diagnosis

To increase this sense of belonging, the information should also be from people who are in the same age group and living the same reality as them:

Me versus someone who is 18 years old, who tells me that it has happened to them before […], versus someone who tells me that as a 45-year-old, they did that. [...] Maybe it’s not the same reality, maybe it’s not the same management [...]. So I’m gonna trust more people of my age.

Participant 7, female, aged 17 years, 14 years since diagnosis

#### Practicality of the Information

Information is considered practical when it is directly related to a real-life situation that participants can identify with and goes beyond theoretical knowledge. Participants are looking for a resource that will “help [them] more with the practical aspect of everything than with the theory” and “that would [...] support them in a follow-up, because of course the lessons are very good, but in the end, [...] the practical aspect [...] is most important [...]” (Participant 21, male, aged 24 years, 10 years since diagnosis).

Information related to blood glucose management and the choice of medical technologies, devices, and suppliers were of high interest. Participants expressed that they should live with a situation to find a use of the information. The use of an insulin pump was given as an example by 1 participant:

I don’t have a pump. Anything that is linked to the pump? No. [...] I’ll just tell you [that] what didn’t happen to me, it looks like I’m not interested [in].

Participant 17, female, aged 23 years, 10 years since diagnosis

#### Novelty of the Information

The novelty of the information refers to the idea that the educational content should provide information that was not known to the participants previously or that the information cannot be found in other places (eg, HCPs, family and friends, and pharmaceutical companies). According to many participants, the amount of unknown information seems to be inversely related to the duration of T1D. For the same reason, participants inquired about having the function of finding specific information in a convenient way (eg, using a search bar or with a hashtag of the keywords) to avoid losing interest in the digital tool, especially for people with a longer diabetes duration. In addition, it was suggested that the tool does not only include “basic topics that can be found on the internet, but that pushes the questions a little further” (Participant 7, female, aged 17 years, 14 years since diagnosis).

## Discussion

### Principal Findings

This study explored the barriers and facilitators encountered in DSM in the context of health care transition by youth living with T1D and adaptation (feature and content) to an adult self-guided DSME/S web app by connecting needs expressed by youth in their DSM with the BCW [18] and its related BCTs [19]. The user-oriented approach used in this study aligned with the recommendations from *the Lancet and Financial Times Commission on governing health futures 2030: growing up in a digital world*, where youth should voice their needs and be placed at the center of the digital health tool development [27]. Having the end user as the primary expert can also increase its usability [28]. In our study, participants highlighted that the features and characteristics included in the self-guided digital tool should facilitate access to information and increase social support and engagement with the tool. The content provided should be reliable, practical (adapted to reality), and novel.

### Simplicity in Finding Information

Barriers encountered by youth in their physical and social environments can often be perceived as uncontrollable and decrease their physical and mental opportunities for performing DSM behaviors. For instance, the “enablement” intervention function of the BCT should be a primary consideration when designing a web-based DSME/S tool, as participants in this study needed easy access to information owing to their variable schedules. Information categorization, short videos, progress saving, and smartphone compatibility are all potential features and characteristics to decrease this barrier and make both the tool and its content available at the convenience of the participants.

### Importance of Receiving Support

The social environment also includes interaction with others, which can imply barriers such as stigma and a perceived lack of social support. Due to these factors, participants reported that they might experience a decrease in their level of confidence in managing their diabetes, make decisions based on the attitude of others, or have fewer opportunities to access DSM-related information. To address these concerns, the BCT “social support” can be used and translated into features such as discussion forums, chat rooms, and shareable links of information from the digital tool. These exchanges provide opportunities to bridge the gap of understanding between youth living with T1D and their family and friends who do not live with this condition, raising awareness of the realities of living with T1D and decreasing stigma and the fear of being judged by others.

In addition, the BCT “feedbacks” could be another integration to decrease external stigma and increase social support while increasing access to information. This technique can be combined with features relating to “social support” (eg, providing feedback for a discussion forum post”) or be used alone (eg, answers given after quiz completion); it can be given in a text format (eg, “Congratulation for your good answers!”), rewards (eg, points provided with the number of quizzes completed), communication with the health care team (eg, providing a medical certificate for program completion), or individualized communication (eg, follow-up phone calls). However, although this method may be effective in achieving self-management outcomes, it may not be feasible owing to financial constraints to produce the feedback algorithm in all circumstances [21]. In addition, feedback must be provided by a qualified person, which further increases the cost of human resources. When comparing feedback via phone calls versus a discussion forum, scheduled calls may not provide the spontaneity that a discussion forum can allow, thus increasing the risk of forgetting the inquiry or losing interest in the matter. The acceptability of this technique varies according to the context. For instance, providing results after a quiz can be psychologically associated with academic performance and becomes a barrier to the use of the related feature. Therefore, it is important to further investigate the use of feedback as a BCT in different groups and its most suitable format.

### Enabling Self-identification

The presence of a role model (intervention function: “modeling”) can increase self-regulation in early adolescence [29] and impact motivation [30]. Associated techniques include “identification of self as a role model” and “social support” and can be brought about by the feature “testimonials.” Participants could have the opportunity to become a mentor for others or be able to identify themselves in the stories of others. Other formats of providing social support in this population demonstrated by the literature include creating teams on the platform using a participant messaging system and the option to share content on social media [31]. However, despite the spontaneity provided by these social groups [23], it is essential to consider the confidentiality of users, especially for the discussion of stigmatizing topics [24]. Therefore, an option of posting information in an anonymous manner should be provided.

### Adapted Content From Credible Sources

Although some DSM factors can be modified by the general design of the digital tool, others such as self-efficacy, diabetes literacy, and access to medical information are directly related to the learning content [32] and the intervention function “education.” The BCT “credible sources” should be integrated to increase the quality of information. In this study, participants distinguished “credible medical source” versus “credible practical source.” The first one often refers to information from HCPs and governmental and diabetes-related organizations, whereas the second refers to information related to daily issues coming from peers living in a similar situation. Therefore, the digital tool should be adapted with the help of different stakeholders to ensure the diversity and credibility of the information. In addition, the BCT “instruction on how to perform a behavior” can be referred to when the information is related to a behavior change; the format of the demonstration (eg, with a real person, in a cartoon, or in a video) can vary depending on the topic.

### Consider Tangible Rewards

Learning content targeting the needs of the population alone might not be sufficient to ensure adherence to the digital tool and to maintain user motivation. As reported by our participants, the lack of motivation for DSM and in using digital tools can be addressed with the use of the BCT “goal setting” [11] associated with the intervention function “persuasion.” This can be translated into a “goal setting” feature within the tool development. The goal can come from the participants or be provided by their health care team. In both cases, the goal must be realistic and attainable objectives. Similarly, a few studies investigated the use of feature “rewards” and “gamification” on the lack of motivation for disease management and digital tool use, this feature was expressed as healthy-living challenges [33,34] team competitions, a points system with monetary rewards [31], and trivia questions [34]. Controversial results were found [35,36]. These differences might be related to the type of reward given and the age of the population. For instance, the participants expressed that the rewards might be a motivator for children but not for teenagers and suggested a preference for tangible rather than virtual rewards. As incorporating features related to gamification (eg, virtual rewards) and a greater level of interactivity are associated with a higher financial cost at the design phase of the digital tool [21] and tangible rewards imply long-term financial investment, we suggest investigating the preferences of the users on these features during its planification.

### Limitations

A few limitations were present in the interpretation of the results. Not all questions were open ended during the interviews. Closed-ended questions were used to validate some concepts and might have biased participants’ answers. To reduce the risk of bias by the researcher when conducting the interviews, the same interviewer was present for all interviews and was asked to follow an interview guide. Translation of the interviews can increase bias in the reporting of the information; therefore, 3 bilingual researchers (LFX, AH, and RC) reviewed the translation independently to ensure translation accuracy. Most participants were White, which could limit the generalizability of the results. The geographical locations of the participants were not recorded during the interviews. Owing to the early exploratory nature of this study, participants provided an overview of their opinion on the included features within the limited time of the interview. Further investigation into how youth would access and use these features is needed.

In conclusion, our analysis demonstrated that youth have an interest in a self-guided digital resource for their DSM where they can encounter peers living in similar situations and who can share their experiences. To increase the sense of support from their family, participants also suggested including sharable links for the information contained in such tools. Given the interest in youth for a self-guided digital tool for DSM, as a future direction, a prototype will be developed and exploration of youth opinion via think-aloud and focus groups will be conducted.

## Appendix

Multimedia Appendix 1 Interview guide.

Multimedia Appendix 2 Example of quotes for diabetes self-management.

Multimedia Appendix 3 Topics proposed by participants.

## Abbreviations

BCT: behavior change technique
BCW: Behavior Change Wheel
BETTER: Behaviors, Therapies, Technologies and Hypoglycemic Risk in Type 1 Diabetes
DSM: diabetes self-management
DSME/S: diabetes self-management education and support
HCP: health care professional
T1D: type 1 diabetes
