# Supplementary material for: Adaptation of an Adult Web Application for Type 1 Diabetes Self-management to Youth Using the Behavior Change Wheel to Tailor the Needs of Health Care Transition: Qualitative Interview Study
Source: JMIR Diabetes. 2023 Apr 26;8:e42564. doi: 10.2196/42564 (PMC10173038; doi:10.2196/42564)
Supplement: Multimedia Appendix 1 [file diabetes_v8i1e42564_app1.docx]

## Multimedia Appendix I: Interview guide

Hi, my name is ________, I am a research assistant working on adapting an online education platform, named *Support*, for youth with type 1 diabetes transitioning into adulthood. This interview should not take longer than an hour. Is that okay with you? (*Confirming consent).*

**PART A: Background information/ Use of online programs**

1. Tell me a little about yourself, how old are you? What grade are you in? How old were you when you were diagnosed with type 1 diabetes?
2. What is your current insulin regimen?
   1. Do you feel comfortable managing your diabetes on your own?
3. Do you ever have questions about your diabetes management?

If yes, where do you go for information?

- 1. *Probe for preferences using online platforms.* Do you use any online programs, websites, or apps for support?
  2. *(If yes)* Which one? What do you like about it? *Probe for details.* How does it help you with managing your diabetes?

1. What do you look for in a website or online platform? *(can be unrelated to diabetes)*
   1. What features do you like on online platform motivate you to continue exploring a platform?
2. What do you think would be most useful in an online support platform that aims to help young people manage their diabetes?

**PART B: Feedback on the *Support* platform**

In the email you received from our team, there was a PowerPoint presentation and a short video about the functionalities of the adult *Support* platform. Did you have a chance to go through that? (*Verifying that they viewed the PPT and or Video. If not, review them with the participant).*

1. Do you have any questions about the adult *Support* platform?
   1. *(If yes) Answer as best as possible, noting down the question.*
2. What did you like the most?
3. In terms of the content of the modules *(a list of topics will be provided with/in the PPT)*, what topics are most relevant to you?
   1. What topics are less relevant to you?
   2. Were there any topics missing that you think is important to include?
4. How do you think the adult platform could be modified for adolescents/young adults?
   1. *Probes:*
      1. Was the language easy for you to understand?
      2. Did you like the look of the platform?
      3. Did you like the features (ex: trophy, certificates…)?
5. Do you have any other comments or feedback to help us adapt the adult platform for young people with diabetes transitioning into adulthood?

**PART C: New content**

To help us get a sense of new content we should create for adolescents/young adults, I will now ask you a few personal questions about how you manage your diabetes.

1. As a young person living with type 1 diabetes, what are you currently struggling with?
   1. *Possible probes:*
      1. Medication regimen? Adjusting insulin?
      2. How to use continuous glucose monitors/insulin pump/technology?
      3. Physical activity/sports?
      4. Disclosing diabetes status to peers/significant other?
      5. Alcohol, drugs, parties?
      6. Stress management, exams?
      7. Managing your diabetes at school/work?
         1. *Probe for details.* Tell me more about this. How do you deal with these challenges?
2. What topics related to diabetes management do you think need to be discussed more openly?
3. Are there any topics that you feel uncomfortable discussing with your doctor, but would like to receive information online, anonymously?
4. What are (or were) your expectations/concerns about transitioning to the adult healthcare system?
   1. *Possible probes:*
      1. Meeting a new doctor?
      2. Dealing with insurance?
      3. Changing pharmacies?
5. What parts of navigating the adult healthcare system do you feel that you would (or would have) benefit from some extra support?
6. What procedures/processes do (or did) you know nothing about and would like (or would have like) explained clearly?

**PART D: Close**

1. Is there anything else you’d like to share? Anything that we missed?

Thank you for your time and your willingness to discuss these issues. Your feedback is valuable and will definitely help us develop a support platform for others like you!
