# Supplementary material for: Adaptation of an Adult Web Application for Type 1 Diabetes Self-management to Youth Using the Behavior Change Wheel to Tailor the Needs of Health Care Transition: Qualitative Interview Study
Source: JMIR Diabetes. 2023 Apr 26;8:e42564. doi: 10.2196/42564 (PMC10173038; doi:10.2196/42564)
Supplement: Multimedia Appendix 2 [file diabetes_v8i1e42564_app2.docx]

## Multimedia Appendix II Example of quotes for diabetes self-management

| External barriers | Codes | Example of quotes |
| --- | --- | --- |
|  | Not being given opportunity for diabetes self-management from parents | “What you want is to be independent. With some parents who can be a bit more overprotective or who are used to being very involved in their children's lives because since they were young, they took care of their diabetes, it can be tough. You can feel a little handicapped if I can say that.” *(Participant 10, female, 19 y.o., 3 years since diagnosis)* |
|  | Complex medical administrative management | “I would say that my concerns are a bit of the unknown. It's because I've been in the pediatric environment since I was 3 years old, I know it by heart. […] I'm going to move forward with someone I don't know […] Who is taking care of me? who is responsible? You will find a doctor; will it be me or my doctor who transfers my file?” *(Participant 7, female, 17 y.o., 14 years since diagnosis)*  “I would say, mostly, about procedures like meeting your doctor. […] Will we have the same frequency and same level of support that we have now? Are we going to have the same resources? […] Is it going to be as accessible as it is right now?” *(Participant 14, female, 16 y.o., 5 years since diagnosis)*  “For me, it would be more about the administrative aspect. What are the programs that we would have access to, then the criteria, what is important to have your doctor fill out when you meet him? […]” *(Participant 3, male, 22 y.o., 6 years since diagnosis)* |
|  | Stigma | “If sometimes you show up with your insulin, I can't even imagine people who have a pump. You show up with insulin then there are people who look at you like ah is that person drugged, what is it? You know they called me junkie sometimes and I laughed. But sometimes it's tiring." *(Participant 4, female, 24 y.o., 10 years since diagnosis)*  “It's when, people don't seem to necessarily know what diabetes is […] They only know that ah you take too much sugar or you can't eat sugar. That's what their idea \|is of you], but they also don't realize that it affects everything, your whole life. Literally everything, everything, everything, everything, everything affects it too.” *(Participant 5, male, 23 y.o., 17 years since diagnosis)*  “I have often been told Ah yeah, I do not understand why you are diabetic, you seem to eat well then you don’t seem to be very overweight. No, but it's not that.” *(Participant 18, female, 16 y.o., 15 years since diagnosis)* |
|  | Variable schedule | “But sometimes it can be hard, like my routine, it can change. One day I’ll have practice, the next day I won’t, and the next day I’ll have a game. Sometimes it’s hard, like I don’t have a daily routine right now. […] Basically, everything can affect your blood sugar is how I see it. Like stress, eating, sleep, I think anything can, which is really hard.” *(Participant 19, male, 16 y.o., 9 years since diagnosis)* |
|  | Stigma | “Everybody's like oh you got diabetes you can't do this, you can't do that, but no [they] don't know [that]. It's more that." *(Participant 7, female, 17 y.o., 14 years since diagnosis)*  “All the time, [it is] so difficult for me to raise my hand and then tell the teacher I'm not feeling well, and that I need to take some sugar. I feel bad all the time because I don't want to interrupt their class and like I always find it frustrating. Then I tell myself like there is like no choice to say it, as I feel worse all the time, I see it, I feel it since in my head I'm no longer [aware].” *(Participant 14, female, 16 y.o., 5 years since diagnosis)*  “I might be afraid of what the person will think if I say I don't want to talk about [diabetes].” *(Participant 2, female, 23 y.o. 14 years since diagnosis)*  “To understand that we are not always good patients […]. That we are not perfect patients, who take their blood sugar on time, and then they eat a certain number of grams of carbohydrates and that we are patients after all and maybe include this aspect of having to respect things.” *(Participant 21, male, 24 y.o., 10 years since diagnosis)*  “When you are younger, you have less confidence in you and sometimes I was like I will wait to the end of the class to eat something. Otherwise, people will look at me as if you were sick. When you are younger you want to be as other people. So, it happened to me to wait for class to end to treat hypos.” *(Participant 3, male, 22 y.o., 6 years since diagnosis)* |
|  | Felt obligated to answer external questions | “You know what makes me uncomfortable, it’s when I must explain it to someone.” *(Participant 17, female, 23 y.o., 10 years since diagnosis)*  “[Diabetes] is still a part of you. It's a bit like your privacy. Then having to go tell someone else about it, having to explain it to them again when you have been living with diabetes for years.” *(Participant 18, female, 16 years since diagnosis)*  “With diabetes, well, we already live with it every day. We do not want to talk more about it to a person for example, who we are just getting to know.” *(Participant 2, female, 23 y.o. 14 years since diagnosis)* |
|  | Perceived inadequate care and support from the healthcare team | “It was like a shock to me because they [my new healthcare team] were supposed to be a medical specialist. Then he had no expertise of technology in diabetes at all. Then he didn't even look at my blood sugar.” *(Participant 3, male, 22 y.o., 6 years since diagnosis)*  “When I was diagnosed, I was 16, weighed 100 pounds. [the doctor] thought I was type 2. Then he said to me, well you're going to the hospital tomorrow, you know? And all while I was totally in ketoacidosis. […] That’s the case for many doctors.” *(Participant 10, female, 19 y.o., 3 years since diagnosis)*  “There’s a doctor at school but he doesn’t really know anything about diabetes, like I’m sure he knows vaguely but not really, so just not having that support and kind of having to deal with it yourself. That’s the hardest thing.” *(Participant 19, male, 16 y.o., 9 years since diagnosis)*  “But I imagine that we will certainly be more to ourselves because, I mean, we are adults, we still must know how to manage our diabetes well. But of course, I would be afraid. In addition, like having less resources [in adult care]. That quite stresses me." *(Participant 14, female, 16 y.o., 5 years since diagnosis)* |
|  | Perceived inadequate support from family and friends | “It's like the world isn't really going to pay attention to “are you okay? Were you in depression? Are you too much in isolation?” […] Sometimes I have heard like yes, but it's a disease like any other disease, yeah well there is a worse disease in the world, stuff like that. […] People around can just pay more attention directly when someone has been diagnosed, just pay a little more attention to that person, because for sure for some, it is easier than for others, and for other it’s more difficult.” *(Participant 8, female, 20 y.o., 6 years since diagnosis)* |
|  | Lack of motivation | “Then it took a little while [after the diagnosis] before I wanted to hear some stuff about diabetes, [...], then maybe a little bit of the stuff that might help these people with diabetes acceptance and take responsibility. I think it would have been fun to talk about it a little more when I was young, to have cues to deal with diabetes […] to be able to explain it without living in too much discomfort, so to speak.” *(Participant 17, female, 23 y.o., 10 years since diagnosis)*  “Since I know how to calculate my carbohydrates, I know everything to do, but sometimes it's to take the initiative, calculate […] it’s more doing it than knowing it.” *(Participant 18, female, 16 y.o., 15 years since diagnosis)* |
|  | Resistance to cooperate with his/her own behaviour | “They’re habits that we have so it becomes more and more difficult afterwards, to break this habit.” *(Participant 4, female, 24 y.o., 10 years since diagnosis)*  “What is most difficult for me is to be consistent [in my diabetes management]. […] to stay consistent over a few months is a bit difficult.” *(Participant 21, male, 24 y.o., 10 years since diagnosis)* |
|  | Mental burden | “Especially on social media, we want to have a beautiful appearance. When we hear that someone is diabetic, it is very difficult because firstly, you know, when we have hypo, at a certain moment you must eat. Of course, it’s more difficult, especially the weight, and especially at our age, to have a stable weight then in addition being diabetic, I find it even more difficult.” *(Participant 14, female, 16 y.o., 5 years since diagnosis)*  “It is something that we need to show publicly because we don’t have any choice. In the sense that if I feel that I am in a hyper, I have no choice than to sting myself. ” *(Participant 17, female, 23 y.o., 10 years since diagnosis)*  “Above all, I would say that the discipline is most difficult. It is a 24/7 disease [diabetes], 356 days a year.” *(Participant 15, male, 21 y.o., 6 years since diagnosis)*  “For sure sometimes it’s more difficult because I mean, it’s like decisions that you constantly make, [...] every moment of the day.” *(Participant 14, female, 16 y.o., 5 years since diagnosis)* |
|  | Support from peers living with T1D | “Talk [to youth living with T1D] about the little things in our daily life that we can’t talk to anyone else about. Well, we could, but who wouldn’t understands as much.” *(Participant 10, female, 19 y.o., 3 years since diagnosis)*  “The doctor, he’ll give me advice. […] But It’s still easier for him to say it as he doesn’t live the same reality as me, but you know to see a little more how people can apply it, sometimes it helps me when I meet a diabetic person.” *(Participant 17, female, 23 y.o., 10 years since diagnosis)*  “A big part that helped me too with diabetes in general is that I went to the Carowanis Camp, where I met very good diabetic friends too. We are a group of 4 females together. When I have small questions, for example the sensibility factor, to increase or decrease it. When I ask the question, I will get an answer. So, exchange on these small things, advice like this, and it helps me a lot.” *(Participant 18, female, 16 y.o., 15 years since diagnosis)* |
| External facilitators | Access to medical therapies and technologies | “With new technologies, for me that […] just between the time I was diagnosed and today there has already been a good difference there.” *(Participant 22, male, 22 y.o.13 years since diagnosis)* |
|  | Access to medical information | “I think that if there is a way to acquire information more easily, more quickly, because an appointment with an endocrinologist is still long. So? I found it would be interesting to consult an application like this [*Support*].” *(Participant 12, male, 14 y.o., 6 years since diagnosis)*  “Knowing that there are professionals who verify the articles. Then all that, it reassures me.” *(Participant 3, male, 22 y.o., 6 years since diagnosis)* |
| Internal facilitators | Diabetes literacy | “It has only been 6 months that I know we can look at the injected insulin dose on it [the insulin pen] and I am very excited. I can know how long it was since I injected my last dose [from the insulin pen]? I didn't know we could do this." *(Participant 4, female, 24 y.o., 10 years since diagnosis)*  “I don't calculate my carbs down to the nearest carb, let's say. I go more by estimate, it is a method that I have had for several years. Then sometimes I question myself and repeat to myself all the time that I should go back to the good old, well, the good method. […] Am I doing this right? Is it enough to avoid having complications later to have a good life, let's say, without it being too burdensome?” *(Participant 7, female, 17 y.o., 14 years since diagnosis)*  “If I didn't inject, what would it do? At 10 months of diabetes, I still don't even know what it does. It's something to know what the consequences are. […] We talk a lot, do that that that that, but you know, if we do not do that, then what will it do?” *(Participant 11, female, 16 y.o., 1 years since diagnosis)* |
|  | Perceived support by family and friends | “I tend to talk less about it [my diabetes] because I don't want people to associate me with that only.” *(Participant 13, female, 16 y.o., 5 years since diagnosis)*  “My coaches also had to understand that OK, we have to give her more breaks, she really has to have some time to adapt before we can go at the same pace we did before”.” *(Participant 8, female, 20 y.o., 6 years since diagnosis)*  “I think making everybody know it [you have diabetes] was the first step. After that, there is no more danger. You will not be judged. Everybody will be okay, he is diabetic, and it is all”. *(Participant 3, male, 22 y.o., 6 years since diagnosis)*  “When you are young, you really need to not feel judged, then, to know that well okay, it’s okay you did your best even if you have a hyper because you ate, I don’t know, a cake, because it was the birthday of someone”.” *(Participant 4, female, 24 y.o., 10 years since diagnosis)*  “For me, it wasn’t a surprise initially because since very young I was watching my father making self-injections. And my sister, who is older than me, was diagnosed with diabetes when she was in elementary school.” *(Participant 15, male, 21 y.o., 6 years since diagnosis)*  “You have to prick yourself […]. It might bother the world, but you don’t care. That’s what you need, and you know you’re doing nothing wrong.” *(Participant 22, male, 22 y.o.13 years since diagnosis)* |
|  | Self-efficacy | “[The blood glucose variation] is hard sometimes, it’s just so unpredictable. […] So sometimes just being on it all the time is the best thing and there’s no one way to deal with it.” *(Participant 19, male, 16 y.o., 9 years since diagnosis)*  “I think I accepted my disease because I never really realized that it’s a disease, because for me, it’s normal.” *(Participant 18, female, 16 y.o., 15 years since diagnosis)*  “Having difficulties to manage your diabetes should not be something that you feel embarrassed about. It is not just a lack of motivation, there are other things. It is a chronic disease that you need to manage every day.” *(Participant 18, female, 16 y.o., 15 years since diagnosis)*    “For sure the mental health aspect is an aspect that is very important for a lot of diabetics.” *(Participant 16, female, 19 y.o., 9 years since diagnosis)* |
|  | Awareness of potential medical complications | “All related to the understanding of why there are complications. Why there is this, why be careful? It is to understand: the why and the how.” *(Participant 18, female, 16 y.o., 15 years since diagnosis)*  “There were like really all the aspects that, that I wondered about, like for me personally like ketoacidosis, that I never really necessarily understood, really detail by detail, what is it.” *(Participant 14, female, 16 y.o., 5 years since diagnosis)*  “Am I going to be able to do the same job [as people who don’t have diabetes] do? They tell me yes, but you know […] they ’on't need to watch blood sugar every half hour.” *(Participant 11, female, 16 y.o., 1 years since diagnosis)* |
|  | Perceived adequate care and support from the healthcare team | “When I was contacted for my first appointment at the adult hospital, [..] I was really told right away where to go, how to proceed. I felt very comfortable to change [healthcare team].” *(Participant 2, female, 23 y.o., 14 years since diagnosis)*  “I have the impression that my doctor knows type 1 diabetes well. […] I was really happy because I have had experiences with less kind doctors about diabetes.” *(Participant 10, female, 19 y.o., 3 years since diagnosis)*  “I found my healthcare team formidable. Honestly, I cannot ask for better. So personally, I have no discomfort in bringing up any subject with them.” *(Participant 14, female, 16 y.o., 5 years since diagnosis)* |
