# Supplementary material for: Adaptation of an Adult Web Application for Type 1 Diabetes Self-management to Youth Using the Behavior Change Wheel to Tailor the Needs of Health Care Transition: Qualitative Interview Study
Source: JMIR Diabetes. 2023 Apr 26;8:e42564. doi: 10.2196/42564 (PMC10173038; doi:10.2196/42564)
Supplement: Multimedia Appendix 3 [file diabetes_v8i1e42564_app3.docx]

Multimedia Appendix III: Topics to be included proposed by participants

| **Topic** | **Sub-topic** | **Example of titles** |
| --- | --- | --- |
| Diabetes-related management | \| - Medical technology management \| \| --- \| \| - Medication management \| \| - Relationship with healthcare team \| \| - Transition to adult care (pre, during, and post) \| | - How can I correct hypo/hyperglycemia?  - How can I keep my blood glucose sensor from falling?  - How can I prepare for my transition to adult care?  - How to establish a good relationship with my new healthcare team?  - How to manage and select my pump parameters?  - How to organise my file transfer?  - How to select my insulin pump?  - List of medical exam recall (e.g., when to do the eye exam)  - List of questions to ask to the doctor during my first meeting at adult care  - What are my responsibilities to ensure a fluid transition?  - What are the different types of insulin and their active period?  - What should I do if I want to change doctor?  - What to do if I give myself too much insulin? What to do if I miss an insulin dose?  - What to do if I have ketone bodies and I am not at home?  - What will my adult healthcare team look like? |
| Medical coverage | - Insurance  - Special support program | \| - How should I transfer from my parent’s insurance to mine? \| \| --- \| \| - Insurance and bursary programs \| \| - What is covered by the provincial health care system? \| |
| Latest news | - Latest research results  - Social events organized by the diabetes organization | - Advancements in the field of diabetes  - New technologies and proposed therapies  - Postings on current research participant recruitments |
| Lifestyle | \| - Alcohol \| \| --- \| \| - Drugs \| \| - Mental health \| \| - Nutrition \| \| - Physical activity \| \| - Sexuality \| \| - Sleep \| \| - Social event \| \| - Travel \| | \| - Carbohydrate counting with and without pumps \| \| --- \| \| - Diabetes and mood swings \| \| - How should I prepare for pregnancy? \| \| - How to prepare for Halloween? \| \| - How to travel with diabetes? \| \| - Recipes and their nutritional content \| \| - What is the impact of physical activity on glycemia? \| \| - Which food should I eat before doing physical activity? \| |
| Pathophysiology of diabetes | - Difference between different types of diabetes  - Etiology of diabetes (especially of type 1 diabetes)  - Medical complications | \| - Are hyperglycemia less serious than hypoglycemia? \| \| --- \| \| - How to deal with nocturnal hypoglycemia? \| \| - What are the factors that can induce type 1 diabetes? \| \| - What are the potential complications if I don’t manage my diabetes properly? \| \| - Why can uncontrolled diabetes lead to complications? \| |
| Peer experience | - Feedback/review on diabetes-related products  - Management of diabetes  - Personal story | \| - How do others live transition in their life (e.g., when changing school, when going to adult care) \| \| --- \| \| - How do others travel with diabetes? \| \| - How to live with diabetes and coeliac disease? \| \| - Opinion on different types of insulin pumps \| |
| Social relationships and interactions | - Explaining diabetes to others  - Perception of others  - Stigmatization related to type 1 diabetes | \| - How to explain diabetes to my family and friends? \| \| --- \| \| - What should I say when I don’t want to talk about diabetes with others? \| \| - What should we do when we are stigmatized by others? \| |
